# A Gallium Oxide-Graphene Oxide Hybrid Composite for Enhanced Photocatalytic Reaction

**DOI:** 10.3390/nano6070127

**Published:** 2016-07-01

**Authors:** Seungdu Kim, Kook In Han, In Gyu Lee, Won Kyu Park, Yeojoon Yoon, Chan Sei Yoo, Woo Seok Yang, Wan Sik Hwang

**Affiliations:** 1 Department of Materials Engineering, Korea Aerospace University, Goyang 412-791, Korea; seungdukim@gmail.com (S.K.); kooookin@gmail.com (K.I.H.); leeig@kau.ac.kr (I.G.L.); 2 Electronic Materials and Device Research Center, Korea Electronics Technology Institute, Seongnam 463-816, Korea; pwkstyle@gmail.com (W.K.P.); yajoony@keti.re.kr (Y.Y.); ychs@keti.re.kr (C.S.Y.); 3 School of Advanced Materials Science and Engineering, Sungkyunkwan University, Suwon 440-746, Korea

**Keywords:** gallium oxide, graphene oxide, photocatalytic reaction, methylene blue, ultraviolet radiation

## Abstract

Hybrid composites (HCs) made up of gallium oxide (GaO) and graphene oxide (GO) were investigated with the intent of enhancing a photocatalytic reaction under ultraviolet (UV) radiation. The material properties of both GaO and GO were preserved, even after the formation of the HCs. The incorporation of the GO into the GaO significantly enhanced the photocatalytic reaction, as indicated by the amount of methylene blue (MB) degradation. The improvements in the reaction were discussed in terms of increased surface area and the retarded recombination of generated charged carriers.

## 1. Introduction

Various metal oxides have been investigated with respect to their photocatalytic reactions, and the resulting technologies can already be found in self-cleaning and anti-fogging/antibacterial fields [1,2]. These products work via the photo-induced redox reactions of adsorbed materials and/or photo-induced hydrophilic conversion of materials [3,4,5]. The acceleration of the photoreaction occurs on a material’s surface, so the introduction of nano-materials and nano-technologies can significantly enhance catalytic efficiency [6,7,8,9]. Meanwhile, two-dimensional (2D) materials, such as graphene and graphene oxide, have been intensively studied in various areas due to their extraordinary high surface-area to volume ratio [10]. Theoretically, a hybrid composite (HC) made up of a metal oxide and 2D materials could facilitate an enhanced photocatalytic reaction and thus further extend and widen the applications of these materials [4]. Although HCs of several metal oxides and graphene/graphene oxide have been explored [4], an HC using gallium oxide (GaO) and graphene oxide (GO) has not yet been investigated. In fact, GaO is an important metal oxide showing a larger energy bandgap (4.8~5.1 eV) than conventional metal oxides, such as TiO_2_ (3.0 eV) and ZnO (3.2 eV) [11,12]. In addition, GaO has recently garnered more attention for use with power electronics [13] and the extension of their applications to various other areas. In this work, a GaO-GO HC is presented, and its material and photocatalytic reaction properties are investigated. Photo-induced charged carriers are generated from the GaO, and these carriers spread out across the GO, which has a large surface area, resulting in a boost to the photocatalytic reaction. In addition, the GO that attaches to the GaO is able to retard the recombination of generated electron-hole pairs.

## 2. Experimental Section

HCs made up of GaO and GO were synthesized using a conventional hydrothermal method that is often used to produce metal oxide powder [14]. GaCl_3_ (99.999%) was diluted in deionized (DI) water to form a synthesis precursor, and the GO was prepared in the DI water as a solvent. After mixing the GaCl_3_ and GO solutions, ammonium hydroxide (NH_4_(OH) 28% in H_2_O) was also added to maintain a pH balance above 8 since the reaction necessary to form Ga oxide nanoparticles would not occur at a pH value below 8 [15]. All of the processes were conducted in a three-neck round-bottom flask in an ice bath to maintain a temperature of 0 °C. The solvent and HC were separated via centrifugal force at 4000 rpm for 30 min. The collected HCs were then washed and rinsed to remove the ammonia and chlorine residues. Finally, the HCs were dried in a conventional freeze-dryer at −80 °C for 48 h. The formed HCs and GaO were characterized via scanning electron microscopy (SEM) (Busan, Korea), X-Ray Diffraction (XRD) (Busan, Korea) and Raman analyses (Seoul, Korea). Furthermore, the photocatalytic reactions of the HCs were evaluated via the degradation of methylene blue (MB; C_16_H_18_N_3_SCl) at 245 nm of exposure. Multiple 100-mg HCs were added to 100-mL MB solutions (0.3 g/L in H_2_O). The mixed solutions were exposed under a 245-nm mercury lamp (Seongnam, Korea) at 0.4 mW/s for 0 min, 60 min, 120 min and 180 min, respectively. For the absorbance spectrum analysis, 5 mL was collected from each mixed solution, and the absorbance spectrum was obtained as a function of wavelength in the range of 450–800 nm. The entire experiment was conducted in the dark to avoid any light source disturbance.

## 3. Results and Discussion

**Figure 1:**
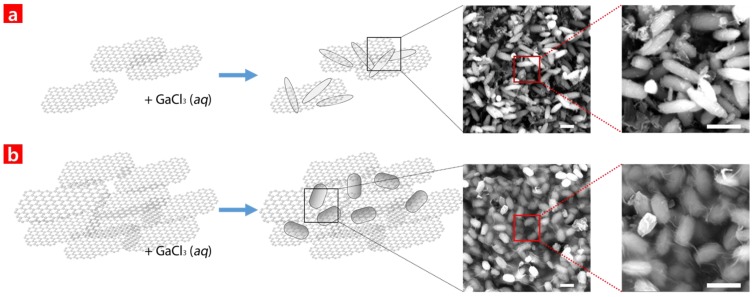
Schematic illustrations of hybrid composite (HC) growth depending on (**a**) a 4% graphene oxide (GO) solution and (**b**) a 10% GO solution. The scale bar in the scanning electron microscopy (SEM) is 1 μm.

The HCs formed via the aforementioned processes are shown in Figure 1. The SEM images in Figure 1a,b shows grain-shaped GaO, and Figure 1b also reveals that the formed GO connected to the GaO in a fishing net configuration. The GaO samples with higher GO concentrations showed higher surface areas, which eventually led to enhancements in the photocatalytic reaction. The GaO only sample was made using a 5 g GaCl_3_ solution. However, the 4% GO-GaO and 10% GO-GaO samples were made using 0.2 g of GO and 0.5 g of GO as well as a 5 g GaCl_3_ solution. In addition, it is interesting to note that the height to width ratio of the GaO with a GO 4% solution decreased in the GaO with a GO 10% solution. It was presumed that the presence of GO might have hindered the GaO growth for a certain structural plane More detailed structure and crystallinity information regarding the GaO and GO was obtained via XRD and Raman analyses.

**Figure 2:**
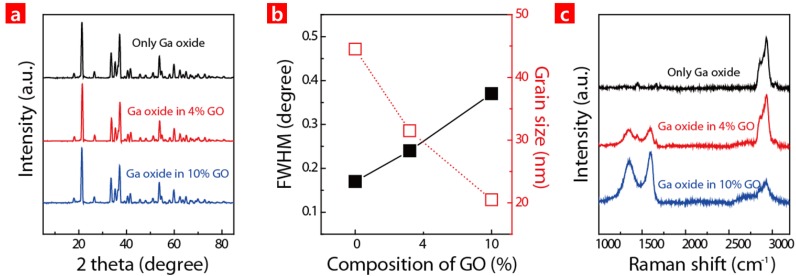
(**a**) X-Ray Diffraction (XRD) spectra of different HCs made up of gallium oxide (GaO) and with/without GO; (**b**) Full-width at half maximum (FWHM) variation for XRD peaks from (**a**) and grain size of the HCs as a function of GO concentration in the solution; (**c**) Raman of different HCs using the same proportions as in (**a**).

Figure 2a shows the XRD spectra of GaO-GO HCs, as well as GaO for comparison. It shows that an orthorhombic structure was preferred in the GaO, and the crystallinity of the GaO was preserved even when forming HCs with GO [16].

In addition, the full-width at half maximum (FWHM) of {110} was measured, and the grain size of the structures was extracted from the FWHM at different GO concentrations [17]. The GaO grain size of 45 nm continued to decrease as the GO concentration increased in the HC. It was presumed that the functional groups such as hydroxyl, epoxide, carbonyl and carboxyl at the GO surface can serve as nucleation sites. Accordingly, there should be more nucleation sites for the GaO when there is a higher GO concentration because this leads to a smaller GaO grain size in an HC [18]. This reveals that the presence of GO during the Ga oxide formation affects the grain size of the Ga oxide in a nano scale range. The crystallinity of the HC was further investigated via Raman analyses, as shown in Figure 2c. The results show that the peaks representing GaO were observed in the GaO particles. As the GO concentration increased in the HC, the GO peak became more prominent. D-band (D) occurs at 1350 region because of oxide. The G-band (G) is generated at 1350 region because of graphite [17]. The D/G ratio of the GO peak in the HC was comparable to that of a conventional GO. The results show that high quality GO was preserved even in the GO in the HC.

**Figure 3:**
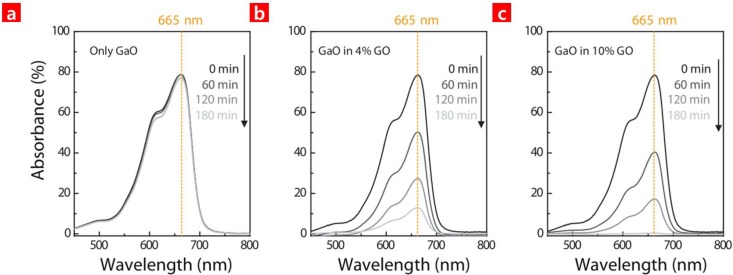
The absorbance spectra of the methylene blue (MB) solution where (**a**) GaO; (**b**) an HC of GaO and 4% GO; and (**c**) an HC of GaO and 10% GO were added to the MB. Each sample was exposed to 254 nm of radiation at different times.

The photocatalytic reaction of the GaO with/without GO was investigated through the photo-degradation of the MB under UV exposure. Figure 3 shows time evolution of the MB absorbance spectrum depending on nanoparticle sample, i.e., only GaO, GaO in a 4% GO solution and Ga oxide in a 10% GO solution. Before the spectra analysis, the samples were exposed at 254 nm for 0 min, 60 min, 120 min and 180 min, respectively. The results show that the absorbance spectrum of the MB with only GaO remained constant. However, the spectrum intensity significantly decreased according to time and GO concentration. This reveals that the MB degradation proportional to the GO concentration, i.e., the total surface area in the MB.

**Figure 4:**
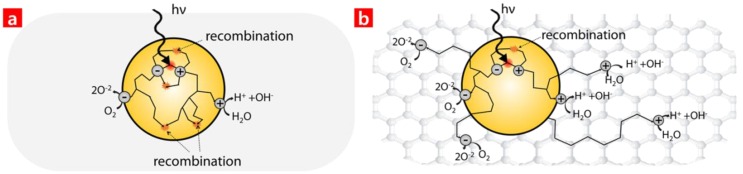
Schematic illustrations of charged carrier generation and transfer, as well as the water splitting mechanism of (**a**) the Ga oxide and (**b**) the HC of Ga oxide and GO. hν is ultraviolet light energy, where h is Planck’s constant and ν is frequency.

A schematic illustration of the charged carrier transfer and MB degradation mechanism of the GaO (Figure 4a) and the HCs (Figure 4b) made up of Ga oxide and GO is described in Figure 4. The HCs made up of GaO and GO were able to slow the recombination of generated electron-hole pairs and enhance the reaction potential with the MB.

## 4. Conclusions

Hybrid composites (HCs) made up of GaO and GO were investigated to facilitate enhanced photocatalytic reactions under ultraviolet (UV) radiation. The improvements in the reaction were attributed to the increased surface area that resulted when the GO attached to the GaO. The generated charged carriers in the GaO under UV radiation spread out through the GO and retarded the recombination of electron-hole pairs, significantly enhancing the photocatalytic reaction with the MB. The material properties of both the GaO-GO were preserved even after the formation of HCs made up of GaO and GO. The HCs developed in this study can be used in various applications requiring enhanced photocatalytic reactions.
